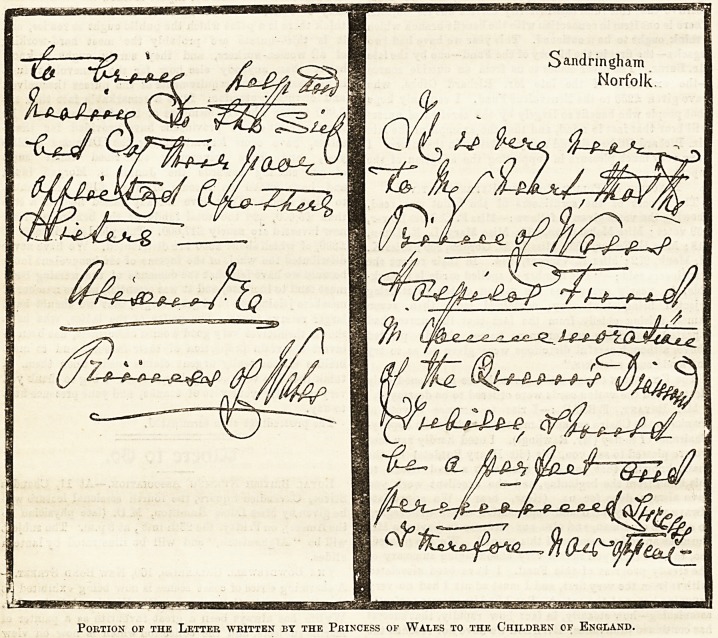# "The Hospital" Nursing Mirror

**Published:** 1898-03-26

**Authors:** 


"The Hospital" Nursing Mirror.

Being the Nursing Section of "The Hospital."

[Contributions for this Section of "The Hospital" should be addressed to the Editor, The Hospital, 28 & 29, Southampton Street, Strand, London, W.C., and should have the word "Nursing" plainly written in left-hand top corner of the envelope.]

### News from the Nursing World.

### IRISH COTTAGE INDUSTRIES.

The exhibition of the Irish Industries' Association was opened at Lansdowne House, Berkeley Square, by the Duchess of Connaught on the 17th inst. The rooms were thronged with distinguished guests, and Her Majesty purchased largely by proxy. The Duchess was accompanied by the Duke, and attended by Colonel Ellis. After pronouncing the exhibition open, Her Royal Highness visited each stall, and displayed great interest in the many beautiful articles exhibited for sale. The stall-holders were ladies who have fostered handicrafts of one kind or another amongst the Irish peasantry. For instance, the Barons Court cottage industry is the knitting of woollen socks for the army, and has for sponsor the Duchess of Abercorn. Art needlework is also encouraged. The sale was as successful this year as last, realising £2,275, but the educational and economic value of the work cannot be estimated by pecuniary results alone.

### EAST END MOTHERS' HOME.

By the kindness of Mr. and Mrs. John Ryder the annual meeting of the East End Mothers' Home was held at 9, Grosvenor Gardens, S.W., Viscount Knutsford in the chair. The past year has been one of growing success to this deserving charity. Funds have come in for all expenses, and also to considerably reduce the debt to the bankers. The Managing Committee, which includes names that are in themselves a guarantee of the good work well done by the home, have wisely secured Dr. Louis C. Parkes as hon. sanitarian. This creation of a new office caused some amusement, and the new officer justified his appointment by reporting upon the sanitary condition of the home. He said that the arrangements were as far as possible good, excepting that he considered that in one ward in the attics more cubic space essential, and that the nurses should not have their dining and sitting rooms in the basement. The committee were able to announce that they had secured the lease of the adjourning house, which is to be fitted up in a way that will meet these objections. The adoption of the report was moved by the Hon. W. F. D. Smith, M.P., seconded by the Hon. Norman Grosvenor, and supported by Mr. Charles Booth, who knows more about the poor in London perhaps than anyone else. The re-election of the committees and honorary officers was proposed by Mr. George J. Goschen, jun., M.P., and seconded by Dr. Louis Parkes. The vote of thanks to the chairman and the hosts was proposed by the Rev. Marmaduke Hare, and seconded by Mr. Carsham Corner.

### HULL JUBILEE DISTRICT NURSING ASSOCIATION.

The Jubilee has had a great influence on the work of the district nurse at Hull. The new movement will be amalgamated with the existing society, and several names added to the committee. A home large enough for eight nurses will be erected on a central site, and any balance after the building and furnishing debts have been discharged will be invested as a reserve fund. Lastly, the name, which has hitherto been the Hull and District Nursing Association, will be altered to the Hull Jubilee District Nursing Association.

### THE NURSES' SITTING-ROOM, POPLAR.

Amongst the gains counted to the Poplar Hospital for Accidents during the last year is the nurses' sitting-room. Up to the time of its completion the dining-room, a cheerful pretty room served all purposes, easy chairs and a comfortable lounge round the fire forming a cosy corner still in favour with nurses off duty. The newly decorated room is artistically arranged. Two-thirds of the walls are wainscotted with lincrusta, which, with windows, overmantel, and bookcases, is painted white. The upper third of the wall is papered with green flock paper, showing up to advantage two charming paintings of children. These and the ornaments on the mantelpiece are the gifts of the chairman, who initiated the idea and actively co-operated with the matron in carrying it out. Mirrors are placed in the panels of the window, and thus add to the light; whilst a dark green pile carpet is on the floor, plenty of easy chairs, a writing-table provided with paper, &c., a couch, piano, and books all help to make this room a delightful retreat. It has been possible to fit up the nurse's sitting-room so perfectly because of what is known as "The Nurses' Luxuries." This fund supplies extras of all kinds for the nurses' use. For instance, two bicycles were added last year as well as many of the plenishings in the newly-acquired sitting-room. The pleasure of the Poplar nurses in their leisure is well catered for. There is a tennis court which is freely used by the more active, whilst hammock chairs under an awning on the terraced roof give rest to the languid out of doors in fine weather. There is another fund which deserves mention. It is the Poor Patient Fund, from which grants are made to the families of disabled breadwinners. It is distinct from the Samaritan fund.

### MISS FARREN'S BENEFIT.

The magnificent sum of over £6,000 was raised by a "benefit" at "Drury Lane," of unprecedented success, on behalf of Miss Nellie Farren, who for twenty years has been a famous burlesque actress. Crippled four years ago by rheumatism, and financially ruined by an unlucky speculation, her professional friends devised this means of relieving her pecuniary embarrassment. The money will be invested by Messrs. Rothschild, who guarantee her in return a handsome annuity with permission to will two-thirds as she chooses, the remaining third being then devoted to charity. Probably £1,000 will endow a "Nellie Farren bed" at a children's hospital for the offspring of actors, and the other £1,000 will be divided between the two larger theatrical charities.

### QUEEN VICTORIA'S JUBILEE INSTITUTE FOR NURSES.

Grosvenor House was the selected meeting place of the Executive Committee of Queen Victoria's Jubilee Institute for Nurses. Lord Reay presided. Sir Arthur Arnold moved the adoption of the balance-sheet, and Sir Squire Bancroft seconded it. The Diamond Jubilee resulted in a sum of £136,000, exclusive of £18,894 collected for local institutions affiliated with the national headquarters. Of the former sum, England and Wales was awarded £29,281; Scotland, £9,134; and Ireland, £1,785. The money subscribed in Scotland and Ireland will be spent in those countries. There is further work in store for the committee, therefore it will not dissolve. Her Majesty's disposal of the £70,000 subscribed by the women of England, on the occasion of the fiftieth anniversary of her accession to the throne, has been singularly happy. Not only is the work filling a sorely-felt need, but it provides a career for trained women.

### A DAY OF REST.

The Day of Rest for the nurses of the London hospitals and of the metropolitan district generally will be held at Hertingfordbury on Friday, June 3rd. Canon Burnside again offers hospitality, and the Bishop of Thetford will give the addresses. Particulars can be obtained from the Rev. A. G. Locke, St. George's Hospital, S.W.

### METROPOLITAN NURSING ASSOCIATION.

The annual meeting of the Metropolitan Nursing Association was held at Grosvenor House, Park Lane, on the 22nd inst., Mr. Henry Bonham Carter (vice-chairman) presiding. This is the principle centre for training nurses for Queen Victoria's Jubilee Institute for Nurses in district work, and during the year 29 nurses were trained for this work, and over 1,204 cases attended. The presence of the nurses in the district has been of considerable educational value to the poor on sanitary matters.

### THE FIRE AT MODYKHANA.

The *Times of India* gives a thrilling account of the disastrous fire which destroyed half the plague hospitals at Modykhana, Bombay. A spark from a small shed at the back of the European Hospital fell on the Jowlee roof of one of the huts, and almost immediately the whole place was in a blaze. Carried by the breeze the flames spread from one group of buildings to another, and amidst the smoke and flames was heard on all sides the cry "Nurse, save me!" The cry was heroically responded to, and with strength called forth by the extremity of the need, the nurses literally gathered their patients in their arms and fled to a place of safety. Miss Snowdon, burdened by two patients, was knocked down by a third patient flinging himself upon her. Everyone worked at the highest possible pressure, and thanks to the zeal and the good order that prevailed no life was lost in the flames. Three patients only, already dying, succumbed to the shock. The nurses who so signally distinguished themselves had only been on duty a few days. Their names are: Mrs. Campbell, and Misses Winscombe (in charge), Wood, Campbell, Snowdon, Brown, Fry, and Buckley. The last five ladies have lost all their possessions, and Lady Sandhurst has endeavoured to raise subscriptions to repair as far as possible the loss. Great as was the damage, the possibility, nay probability, of the danger spreading leaves those who took part in the stirring scene thankful that the whole place was not destroyed. As for the nurses who behaved so admirably, not a few will envy them the chance of showing heroism which they so bravely seized, whilst all accord to them in sympathy their meed of praise.

### AMERICAN NURSES FOR CUBA.

Miss Clara Barton, who has undertaken the nursing at Cuba, has so far only called one Red Cross sister to Havana, as she intends training the native women, if possible, for the work. Mrs. Monac Lesser, or Sister Bettina, as she is called, has been Sister Superintendent of the Red Cross Hospital at No. 233, One Hundredth Street, New York, where she has had charge of the education of the district nurses and for those to be engaged in emergency work under the Red Cross rules. Her husband, whom she accompanies to the scene of her new work, is a medical man, as are also the other two members of the party, Dr. F. Eagan, of Boston, and Dr. J. B. Hubbell, of Indiana.

### A VENETIAN TRAINING SOCIETY.

A society for the training of nurses (Scuola per Infermiere) has been started at Florence, under the presidency of Princess Antonietta Strozzi. The aims of the committee, as set forth in the prospectus, are at once philanthropic and practical. It desires to train "for the family and the hospitals nurses capable not only of giving kindly aid to the sick, but of seconding conscientiously and scientifically the work of the physician and the surgeon." The first of a series of lectures in favour of the society was given on March 14th by Professor Grocco, before a deeply-interested audience. English nurses were represented by Miss Turton and Mrs. Belcher Houghton.

### MALE AND FEMALE SANITARY INSPECTORS.

In practice it is found that male and female sanitary inspectors work together in cordial good fellowship. Though the male inspectors have collectively refused to admit women to the membership of their society, the women inspectors are unanimous in their praise of the individual kindness and courtesy of their brother workers—a good comradeship which is invariably the result of a working co-operation between the sexes. In districts where both men and women sanitary inspectors are employed, drainage and construction defects, when discovered by the women, are passed over to the masculine department; while overcrowding and the mere social branches of sanitary inspection are looked after by the women.

### SHORT ITEMS.

The Bristol Nursing Institution has been very busy this last year. Epidemics of typhoid, influenza, and measles followed each other, and during the autumn the nurses have had as much work as they could do. Through all, however, they have enjoyed very good health; one, however, was attacked by typhoid, from which she recovered. Oddly enough she caught the complaint at some distance from the scene of the epidemic. The nurses by their own request have had a blue and white enamel badge given them, in order to distinguish them from other nurses, to celebrate the Diamond Jubilee, and also the founding of their institution in the year 1862.—Nurse Smith, who some time ago founded the West Riding Nurses' Association at Halifax upon a basis giving the nurses a large proportion of their earnings, is exciting considerable attention by a series of lectures on "Sick Nursing." Her audiences increase in number, and the younger generation quickly grasp the value of her teaching. The Bradford School Board have secured her services for a course of lecturing.

### Lectures to Surgical Nurses.

By H. A. Latimer, M.D. (Dunelm), M.R.C.S. of Eng., L.S.A. of London, Consulting Surgeon, Swansea Hospital; President of the Swansea Medical Society; Lecturer and Examiner of the St. John Ambulance Association, &c.

### XXVII.—DESCRIPTION OF AN EPILEPTIC FIT—EPILEPTIFORM CONVULSIONS: WHAT THEY INDICATE, AND THE NURSE'S DUTIES IN OBSERVING THEM.

Although a course of surgical lectures does not, strictly speaking, take in the subject of epilepsy, I shall not hesitate to describe one of the two types of that malady, as, in doing so, I hope to be able to give you a word picture of the convulsive state, on which I can draw for other illustrations of my subject. I do not propose to give you a full description of an epileptic seizure, but shall only use such parts of it as serve my purpose now. In a characteristic attack of epilepsy the sufferer is thrown, as it were, on the ground, by a violent shock, which leaves him there with the greater number of his muscles rigidly contracted; his mouth is twisted aside, his arms and legs are contracted, and his hands are clenched; and, as the muscles of his chest are also in this state of tonic spasm, his breathing is arrested. After this stage has lasted some seconds the contracted muscles relax, and now the fixed and rigid state of the body is exchanged for one in which no limb is at rest, as all of them are being twisted and contorted, in an irregular manner, by clonic or intermittent spasm. And, to complete the picture, when the muscles have regained their ordinary state of quietude, the patient, who has been unconscious from the time he was first seized, often falls into a deep sleep, from which he can only be aroused with difficulty. I call you to notice particularly that an epileptic attack frequently finishes off in the condition of sleep, or in a dazed and only half-consciousness. It is the absence of this phenomenon which marks the difference between ordinary surgical affections of the nervous system—that is, affections which are oftentimes to be remedied by operations—and medical ones; for the convulsive muscular movements in the surgical type do not, as a rule, conduce to drowsiness or unconsciousness. You will often hear the word Epileptiform applied to the muscular spasms I am alluding to; its meaning is "like or similar to epilepsy." I will now inform you in what class of cases epileptiform convulsions are met with, and direct you as to the observations which you must make on them.

When that portion of the brain which contains the centres, or controlling stations, for movement of muscles in the body is irritated in any way, from time to time the stored-up irritation is apparently liberated in a sudden manner, and, that irritation being conveyed along definite tracks of nerves, sets up twitchings of the muscles which these supply with nerve force. If a small portion only of the brain is affected the convulsions following its irritation will be at first limited to the muscles which that part controls, and all the spasms which occur from time to time will commence in the same part of the body, and be limited to that part for awhile before they spread themselves over the muscles elsewhere. There is a reason for this peculiar behaviour, and one which affords a valuable clue to the treatment by telling us in which part of the brain the irritation is seated which is revealing itself in this manner. Physiological experiments have now shown positively that the brain contains a number of "centres," and that, while each "centre" has control over a certain limb, it is also capable of being affected or joined in action with other "centres," also having exclusive control over other limbs. Thus, experiments have shown us that if the same portion of a brain is irritated again and again, a same portion of the body responds by convulsive movement; and that if that portion of the brain is destroyed the muscles of the body which were formerly thrown into a state of spasm are now paralysed and rendered useless, because they no longer receive any supply of nerve-force to set them into action. These experiments, oft repeated with a definite object in view, have resulted in the mapping out of the brain into areas; and as these areas contain "centres" for control over the movements of the leg, arm, face, head, &c., and even extend into such refinements as controlling the muscular action of individual parts in those limbs, such as the shoulder, elbow, wrist, fingers, and thumb, this search for the "localisation of function" has led to some of the most brilliant achievements in modern surgery; for now, if distinctly localised convulsions set in from time to time, and the same part or limb in the body twitches, we know that the "centre" for movement of that limb in the brain is being subjected to irritation; and the operation of opening the skull—called trephining—being performed, the "centre" is cut down upon and the irritating body is removed, and the convulsions cease. In most cases the irritation proceeds from the presence of some body which is pressing upon the brain—a portion of skull which has been driven inwards by injury, lymph or blood-clot, or some kind of tumour. Should it be the latter, it will behave as most tumours do, and, continuing to grow, press upon and destroy more and more of the brain, as time goes on. Sometimes these localised convulsions extend into general ones, so that after the limb which is first and uniformly affected has twitched for awhile, muscles elsewhere in other parts of the body do the same; and during all this time there is no loss of consciousness. The two facts—that the sufferer does not lose consciousness, and that the convulsions always affect the same part of the body to begin with—distinguish these epileptiform attacks from a true attack of epilepsy. As we are largely indebted to Dr. Hughlings Jackson, of London, for our knowledge of these distinguishing points in the two complaints, the variety of epilepsy I have been speaking of is termed "Jacksonian Epilepsy." As nurses, you have to intelligently observe and record the mode of onset of an attack: which limb is moved; whether muscular spasm is limited to this limb, or whether it spreads to other parts; what length of time is occupied by the whole process; and whether perfect consciousness is maintained throughout the convulsions.

### Minor Appointments.

Stranraer Cottage Hospital.—Miss Annie Ford, for the last seven years Queen's nurse at Kirkcaldy, was appointed Nurse-Matron of this hospital on March 18th. She was trained at Truro, and was nurse in Dewsbury Infirmary for some years.

Eastern Hospital, Homerton.—The following have been appointed Charge Nurses at this institution: Miss Birdsall Hobkinson, trained Brownlow Hill Infirmary, Liverpool, and has since worked under the Metropolitan Nursing Association and as nurse-matron of Tetbury Cottage Hospital, Gloucestershire; Miss Cruice, who was trained at the Mater Misericordiæ Hospital, Dublin, and afterwards was head nurse at the District Asylum, Monaghan; and Miss M. J. D. Burrell, who was trained at the Radcliffe Infirmary, Oxford, and was afterwards for two years ward sister at the National Hospital for Paralysis and Epilepsy, Queen's Square W.C. We published these particulars last week, but as through a printer's error Miss Hobkinson was described as Hopkinson, and we have now fuller particulars respecting the other nurses, we reprint the appointments.

### Sick Diet: Milk.

BY A TRAINED NURSE.

(*Continued from page* 200.)

MUCH improvement has taken place of late years in dairies, cowsheds, and the inspection of food generally. But side by side with improved legislation in these respects has grown up an ever-increasing knowledge of how to adulterate without detection. And with the decadence of simple, rural ethics the difficulty of obtaining milk "as the cow gave it" is becoming greater. I like those good old-fashioned days when a thundery atmosphere "turned the milk." For this was at least a sign that the milk for household and family use was not doctored with salicylic or boracic acids, to such a degree as to be harmful to health. There are dozens of devices of a chemical nature in most dairies nowadays to keep milk fresh. And each new "milk-sweetener" invented is so much on the wrong side of the health balance. Any invention which tends to the keeping of milk at all needs discouragement, because milk should be absolutely fresh and new. Keeping on ice is the only harmless method, and even this hygienic "keeping" may easily be carried beyond the limits of healthy dietetics.

We constantly hear of the "degeneracy" of the modern baby, against whom the indictment is brought that he cannot digest the milk that his infantile forefathers took with delight, safe from further consequences of colic and pains. But the question arises whether it is so much the fault of the modern baby as of the modern dairyman, whose knowledge of chemicals is both theoretical and practical. A healthy baby stomach has a right to, and does, revolt against the constant intake of chemicals. The proper feeding of cows is another point which seriously affects the invalid and infant. I have seen the most serious diarrhœal troubles in babies and severe vomiting in adults through the drinking of milk from cows fed on frozen turnips. The milk of the stall-fed cow, too, is most unwholesome, for the day is long past when Dr. Fothergill could state in his picturesque way that "cows have this advantage over man, that they must be brought up in the country." Fortunately there are a few dairies whose milk can be relied upon, and the nurse should make herself acquainted with these. I find many nurses in private practice have acquired the habit—and it must be confessed that many nursing books give them the warrant for it—of adding so much bicarbonate of soda or magnesia to each tumbler of milk for their invalid, with the idea of thus counteracting acidity and a tendency to "milk constipation." Sometimes the soda is added to keep the milk "sweet." In many cases I have seen this custom produce very harmful effects on digestion, and bearing in mind the chemical substances so often already in the milk this is not a surprising circumstance. The nurse should never make the addition of soda or magnesia to milk unless this is ordered by the doctor. Where so many other and healthier means exist for making milk more digestible and preventing acidity it is a pity to resort to drugs to gain this desirable end.

With regard to the dangers of milk—the absorption of disease germs and the adulteration of it by means of contaminated water, &c., the method of sterilisation has stepped in to minimise the risks. But sterilising will have no effect on the chemicals in milk, and there can be no question that this process destroys the anti-scorbutic qualities so valuable and so necessary to a patient living on an entire milk diet. And sterilised milk has lost the pleasant taste of the fresh fluid. This may to some extent be overcome and some of the anti-scorbutic property of milk restored if it be skimmed before sterilisation and the fresh cream added afterwards. I have seen many cases of scurvy among highly-cared for infants who were fed from birth on sterilised milk. Indeed so many cases of this kind have occurred in New York, where milk sterilisation i much more general than with us, that some of the leading doctors there have declared against it, and have gone back to the old method of fresh milk boiled. But the cases of scurvy are doubtless trifles light as air compared with the myriad cases of diarrhœa and digestive mischief caused by raw and perhaps fermented milk. The use of sterilised milk may perhaps be a choice of evils—and one may long for a condition of health when the deadliest germs may be daringly converted by strong healthy constitutions into fine bone and muscle. But that millenium is apparently far off. We must therefore be content in this present stage of civilisation to take advantage of the safeguards afforded by the process of sterilising. At the same time, one can hardly help recognising that the scheme of Nature makes for healthy cows whose milk would be a quite safe drink, rather than towards a confession that the milk of 19th century cows is a more or less poisonous composition, which needs a lengthy dispensary treatment before being a suitable drink.

Dr. Cheadle gives the following table as to the comparative digestibility of various forms of milk, and it seems eminently practical for the nurse to have such a table for reference :—

1. Peptonised milk.
2. Cow's milk with barley water.
3. Cow's milk and lime water.
4. Condensed milk.
5. Cow's milk with bicarbonate of soda.
6. Boiled cow's milk.
7. Fresh cow's milk.

It will be seen that this table applies only to ordinary and easily-prepared forms of milk. The more complex methods of artificially-prepared and easily-digested varieties of milk which may be set before the invalid will be given subsequently. Meanwhile I shall have a few words to say on each of the mixtures of milk mentioned in the table.

PEPTONISED MILK.—As nurses well know, the chief digestive pitfall in cow's milk consists of the firm clot which forms when the acid gastric juice comes in contact with it. To many stomachs the clot is absolutely unyielding, and it is this stumbling-block which arises on the threshold of digestion which has led to the innumerable mixtures with milk of various substances which dissolve or subdivide the resisting clot. Of all measures the process of predigestion by the peptonising process is the most successful. The recipe for this is so universal it is not worth encroaching on space to repeat it. It will suffice to remind the nurse that the peptonising process converts the milk-curd into a soluble peptone, and no clots are formed when peptonised milk comes in contact with gastric acid juices. This is theory. But as a matter of practice it will be familiar to most nurses that some curds do frequently appear in the motions of sick people living entirely on peptonised milk. But such curds are limited and very soft, and the digestive difficulty is thus minimised to the least possible point. The well-known unpleasant taste of peptonised milk is one of the drawbacks, though this may be lessened by scrupulous care in preparation and by the time-honoured practice of adding a little coffee. Peptonised milk should be sweetened with milk sugar, and if skimmed before peptonisation and the cream added afterwards the drink will be much more palatable.

### A USEFUL AMERICAN SOCIETY.

THE Hospital Book and Newspaper Society is one that for upwards of twenty years has supplied the hospitals, prisons, and charitable institutions of New York with literature. Latterly the work has outgrown the pecuniary strength of its organisers, and they have appealed to the public for funds to carry it on, as it is greatly appreciated.

### Nursing in Paris Hospitals.

### A.—LAY NURSES.

### VI.—REGULATIONS, UNIFORMS, AND ALLOWANCES.

ANCIENT as is the organisation of the Paris hospitals into a homogeneous body subject to one administration and to identical rules of government, it is a most curious fact that there is in the establishments themselves the most complete absence of any public announcement of the rules which are supposed to govern them. The visitor walks through miles of wards, courts, offices, and passages, and sees nowhere, as, almost invariably found in other Government departments, any series of posted regulations for the guidance of the employés, the patients, or the public. Here or there some insignificant morsel of a regulation may appear, but this is very rare. Moreover, when one inquires of the officials for a copy of such general regulations, they either profess complete ignorance of the existence of any such code, or declare that the director of each hospital makes his own interior regulations.

One general set of regulations formulated by the Assistance Publique has fallen into such oblivion as to make many of the hospital staff believe that it never existed. The staff seems in each case to follow its daily tasks according to oral tradition, and in case of dispute it is apparently oral decisions of the superior which is invoked, and not any printed law. A reform is, I am told, imminent, however. The regulations, at present buried in a long series of forgotten circulars, are to be revised and codified, and copies issued to the whole hospital staff.

For all that they may be generally ignored, the Assistance Publique possesses a full set of minute directions, detailing the hours of employment and all matters concerning daily routine. These rules only apply, however, to the three grades of upper or matron nurses, and were adopted by the Council of the Assistance Publique August 3rd, 1882. Although these rules are not for the rank and file, and are even for the upper grades so little used, I will summarise them. Of course, they represent in a general way the procedure in the hospitals at present, and copies of them are supposed to be posted in each lodging and handed to each nurse, although the directors seem to take great liberty in varying them at pleasure. The day service is from six a.m. to eight p.m. Where there is a head nurse, and one or more under-heads or assistants, there can be a dog watch from six to eight p.m. The night service is from eight p.m. to seven a.m. Actually they were kept on duty until twelve noon. Of late, however, the night nurses have been allowed to leave the hospitals at ten a.m., and arrangements are being made to permit them to leave at eight a.m. The meal times are as follows: Half an hour for first breakfast, between a quarter-past seven and half-past eight a.m.; one hour for second breakfast, the hour being fixed by each director; one hour for dinner, between five and seven p.m. Each of these meals is taken successively by two sections of nurses. In each establishment a refectory must be furnished to those upper nurses who wish to take their meals there. Upper nurses on day duty may go out daily from eight to eleven p.m.; those on night duty, from two to seven p.m. They may also go out during the hour of second breakfast for household purchases. The upper nurses have one day's leave a week, on condition that they are in at eleven p.m., and have their service previously provided for, and are not absent on public visiting days. Special permits may be given by the directors. No person in the hospital service may be employed as a servant. Servants from outside can only be employed by special permission of the director. Upper nurses must take their meals in their respective lodgings or in the refectory. Their food must not be brought into the wards. They cannot receive visitors when on duty, except with the director's permission. The lodgings must be kept clean; the director will assure himself of this in the interests of the establishment and of general health. Only the husbands and children of nurses of the upper grades may live in the lodgings, except by permission from the Director of the Assistance Publique. Permission for temporary lodging can be given by a hospital chief. Persons authorised to lodge with the upper grades must conform to regulated hours. The upper nurses are forbidden to receive gifts from patients or from the families of patients. They must not take charge of any money, jewellery, or valuables belonging to patients or under-employés. The uniform is obligatory inside of the establishment; it must not be worn outside at night, nor when on leave. This rule applies to all employés lodged.

The uniform of the male nurses is a dark blue suit with a naval cap bearing the letters "A. P." in front. Great complaint is rife about the oppressiveness of this uniform in hot weather. The female nurses wear black dresses with white aprons, long white sleevelings, and small white muslin caps. These caps are replaced in the case of head nurses and assistants by black silk caps.

The yearly allowances in this matter of dress are in most instances absurdly inadequate. Thus, for women's boots the allowance is 6 francs, payable half-yearly. This would never pay the cobbler's bill of the most economical. The men's allowance is only a franc yearly more than that of the women. In the matter of stockings women are allowed one woollen and one cotton pair each year, and men five pairs every two years. The women of the upper grades have a winter dress each year, while all the others have only one in two years. The head nurses have also a summer dress each year. Each woman nurse is given a metre and a half of the same material as her dress for mending purposes. Each woman is given a stuff petticoat yearly; the head nurses are supplied with the material, while the others are furnished ready-made. The male nurses have two cravats each year, the female nurses having two kerchiefs—muslin for the head nurses, and calico for the others. The men all have a waistcoat each two years, and a new cap yearly. The higher grades have overcoats each year, while the lower grades have to wear theirs for two years. The upper grade women have four aprons a year, the lower but two. In case of accident, however, extra aprons are allowed. The upper nurses have four pairs of sleevelings each year; the lower nurses (who have to soil them much more) but two. In fact, the grades which have the most work to do, and the most occasion to soil their clothes, have the smallest allowance. The inadequate clothing allowance is being considered by a Committee of the Assistance Publique just now, and some improvement may shortly be looked for.

For the lodgings a certain amount of linen is allowed. These allowances are all included in the pay of those living outside, but these outside lodgers can procure the various articles of their uniform at cost price at the Magasin Central of the Assistance Publique.

Great complaint is rife concerning the lack of vacations. The hospital chiefs are but meagrely supplied with substitutes; in fact, many of the directors feel much difficulty in furnishing substitutes for nurses absolutely obliged to be absent from their duties, as in the case of sickness, and, when so many are married, of occasional child-births. There is, in fact, a constant criticism that the night-watches are in the hands of incompetent grades, owing to a lack of a proper staff of qualified matrons for this work.

One of the chief ameliorations lately made has been in the matter of food. The Committee appointed in 1896 by M. Peyron, on account of the tuberculosis scare, to examine into the hospital service in this respect, among many strongly-worded criticisms, attributed part of the contagion in the nursing staff to bad food, and the Municipal Council authorised considerable betterment. All is not yet considered satisfactory in this respect, and another Committee is considering further ameliorations in the food supply.

Edmund R. Spearman.

### Nurses for Klondyke.

There are always adventurous spirits who long to rush off to the uttermost ends of the earth as soon as the opportunity is given them, and there are doubtless many nurses who envy those selected by Lady Aberdeen for duty at Klondyke. The number sent will be to a great extent controlled by the amount of money received in response to the appeal which appeared in nearly all the papers a short while since for the "Victorian Order of Nurses' Klondyke Expedition." The first contingent consists of a superintendent and three trained district nurses, and will be despatched as soon as it can be got ready. The superintendent's stipend is fixed at £120 per annum and her assistants' at £100 a year each; they will be provided with tents and sent to their destination free of cost under the charge of the North-West Mounted Police. They will take provisions for a year, and a full outfit of warm clothing. There is little, if any, chance for English applicants, as Lady Aberdeen speaks of the "women she has in view" being well fitted for the work. Still, if there are any who are determined to try their luck, they must apply direct to Lady Aberdeen, the Government House, Ottawa. Already a tremendous number of gold-seekers throng to the new fields, and although doctors are plentiful they are for the most part not in that region for the purpose of practising their profession. Nurses will have plenty of work, attending frost bites, accidents, and possibly fevers, with all the train of maladies that follow the crowding together of human beings without wholesome food and sanitary conveniences.

### Wants and Workers.

Miss E. Austin, Ravendale, Weybridge, has "How to Become a Nurse" and a "Handbook for Women," by Miss Blackburn, for sale second hand.

Can anyone lend a pelvis and fœtal skull to Nurse E. S., 30, Retreat Place, Hackney, for a short time. Greatest care taken, and deposit paid if required.

E. C. P., 8, Victoria Square, S.W., wishes to obtain "The Handbook for Attendants on the Insane." As the new edition will not be ready for some little time she would be glad to hear of a second-hand copy.

Nurse Wardley, 101, Isledon Road, Finsbury Park, London, N., will sell cheaply a large double Thomas splint.

### Our American Letter.

The care of the insane is receiving considerable attention in the United States, and a plan for the training of attendants has been sketched forth in an article by Dr. Ella V. Timmerman in the *Trained Nurse* for March. Her idea is that the school should be away from the asylum, and the pupils, who should belong to the upper classes of society, should not be in the same building with the patients when off duty. The influence of rank and uniform is very great in dealing with the mentally diseased, and Dr. Timmerman considers that by due attention to these points much of the coercion now needed with refractory patients would be avoided.

Miss Sarah G. Whitney, who has been directress of the Roosevelt Hospital for the last five years, has resigned. The senior nurses have presented her with a beautiful pearl pendant and the juniors with silver toilet accessories as a mark of their appreciation.

Amongst several interesting papers read and discussed before the fifth annual convention of the American Society of Superintendents of Training Schools opened last February at Toronto was one on "Hospital Laundries," another on "Diet Kitchens," and others on "How Far are Training Schools Responsible for the Lack of Ethics Amongst Nurses" and "The Superintendent of Nurses," topics bearing very practically upon a nurse's duties.

The Nursing Department at the Health Exposition to be held at the Industrial Building, New York, will be under the management of Miss Mary E. Wadley. Prizes will be awarded for the best kept sick-room record, for the best design for articles used in the sick-room, and for the daintiest invalid tray. Amongst other instructive models will be one of a suite of living rooms for nurses in their training schools.

Miss Maria Keith, M.D., has recently taken charge of the Phyllis Wheatley Sanatorium and Training School for Nurses. This institution was opened some time ago in New Orleans in order to give young coloured women an opportunity of learning nursing, for which profession they are well adapted.

### The Plaistow Maternity Charity.

The annual meeting of the Maternity Charity and District Nurses' Home, Plaistow, was held at Wimborne House, March 21st, the Bishop of St. Albans presiding. The Bishop, in his opening remarks, spoke strongly in favour of the charity, and the subsequent speakers, among whom were Dr. Playfair and the Mayor of West Ham, showed how necessary such an institution was, especially in a district containing so many poor as the district of West Ham. Not only the lives of the mothers but the future well-being of the children were at stake in many cases where the income was far too small to allow any margin for medical fees, consequently children who might otherwise develop into healthy men and women were maimed and wrecked for life at birth.

The nurses at Sister Katherine's Home have three months' training, and are very practical and efficient in their methods, having all such experience as is gained by attending cottage cases, where perhaps a hospital nurse would be somewhat at a loss for some appliance, &c., which she had been accustomed to use. Funds are greatly needed for the charity, for not only did last year's expenses exceed the income, but the new Nurses' Home, which is to be opened in two or three months' time, will require furnishing and also extra funds for maintenance and increase of work.

The resolution that the work could only with difficulty be carried on in the absence of fresh contributions, and more especially annual contributions, which are earnestly requested, was seconded and carried unanimously. The meeting was fairly well attended.

### The Royal National Pension Fund for Nurses.

### THE ELEVENTH ANNUAL GENERAL MEETING.

THE eleventh annual general meeting of the Royal National Pension Fund for Nurses was held at the offices, 28, Finsbury Pavement, London, E.C., on Thursday, March 17th. Pending the arrival of Sir Henry Burdett, K.C.B., who was to take the chair in the unavoidable absence of Mr. Everard A. Hambro, the Chairman, Mr. EDWARD RAWLINGS, presided. Among those present were: The Hon. Egremont J. Mills, Dr. Thomas Bryant, F.R.C.S. (consulting surgeon to Guy's Hospital), Mr. Walter S. M. Burns, Mr. J. Pierpoint Morgan, jun., Mr. G. Norman, Dr. E. C. Perry (medical superintendent Guy's Hospital), Miss F. C. Nott Bower (matron Guy's Hospital), Miss Mable Cave (matron Metropolitan Hospital), Miss Rosalind Pritchard (hon. secretary of the Junius S. Morgan Benevolent Fund), Mr. George King, F.I.A., F.F.A., Dr. George W. Potter, and others.

The SECRETARY (Mr. Louis H. M. Dick), having read the notice convening the meeting, the report and balance-sheet, which had been printed and circulated, were taken as read.

Sir Henry Burdett having been detained at another meeting, Mr. Rawlings took the chair pending his arrival.

Mr. EDWARD RAWLINGS said: Ladies and Gentlemen,—I have to remind you of the very sad loss we have sustained in the death of our late beloved chairman, Mr. Burns, and to regret the absence of his successor, Mr. Hambro, who is at Biarritz. I will not say more now as, doubtless, Sir Henry Burdett, who has been identified with this institution so long, has prepared some remarks which he would like to make both as regards his testimony to our late respected chairman and to the choice of his successor, in which he feels so great an interest. Therefore, I think the better plan will be not to move the adoption of the report and balance sheet until Sir Henry comes.

Dr. Potter and Mr. W. S. M. Burns were then elected to act as scrutineers.

### RE-ELECTION OF RETIRING MEMBERS OF COUNCIL.

Mr. NORMAN proposed the re-election of the five members of the Council retiring in rotation, viz., Mr. Everard A. Hambro, Sir William Broadbent, Bart., M.D., Mr. E. Rawlings, Mr. W. S. M. Burns, and Mr. C. Eric Hambro. He said: Mr. Everard Hambro has succeeded our late lamented chairman, and I am sure he will prove a worthy successor. All the other gentlemen whose names have been mentioned have been of great value on the council. The Hon. EGREMONT MILLS seconded the resolution, which was carried unanimously.

### RE-ELECTION OF AUDITOR.

Mr. RAWLINGS proposed, and Dr. PERRY seconded the re-election of Mr. Frederick Whinney, F.C.A., as auditor, his fee to be 35 guineas. The chairman observed that the auditor's work increased every year, and its growth had greatly added to his responsibilities.

### ADOPTION OF REPORT.

Mr. RAWLINGS moved the adoption of the report and balance-sheet. The resolution is that the report and balance-sheet be received and adopted. It is usual for the chairman to make a few observations on the accounts. I did not come prepared with any remarks of my own, but we all feel very much the great loss we have sustained in the death of our late chairman. It would, however, be very satisfactory to all to know that Mr. Hambro had consented to occupy the chair in his place. In reviewing the work of the previous year, I can only say it has been particularly uneventful, except in the steady increase in the number of new members; 714 policies have been issued, and that figure was above the average. The invested funds had risen from £311,000 to over £372,000, a net increase of over £61,000, exclusive of the funds of the Benevolent Fund, cash at bankers, &c. The value of the securities held by the Fund at the end of 1897 stood very considerably above the cost at which they stood on the books. There was a balance of over £1,000 to the credit of the sick pay branch after ten years' work. A proof that the Fund is carrying out its practical work is very clearly demonstrated by the fact that at the end of last year there were 188 policies under which annuities were being paid, as against 146 in the previous year. During 1898 over 40 would fall due, so that the number at the end of this year would be considerably larger than at present. On every point the members could congratulate themselves on the thoroughly sound and satisfactory state of affairs shown by the report. (Hear, hear.) The quinquennial valuation was due at the end of the past year 1897, and Mr. George King, F.I.A., is engaged in preparing the valuation, prior to the apportionment of the bonus additions. The work entailed is very heavy and laborious, and cannot be completed for two or three months. A supplementary report will be issued as soon as Mr. King has completed his investigations. It is a very important work, indeed, and takes a very long time, but when the result is arrived at it will be exceedingly interesting, especially to those who are recipients of the benefit. An interesting report of the Junius S. Morgan fund has been prepared, and the thanks of the Council are due again to Lady Rothschild as president, Miss Pritchard the hon. secretary, and to Miss Leigh, the honorary lady visitor, and to all the members of the Advisory Committee, for their untiring attention. It gives me great pleasure, and I am very glad, to welcome the grandson of Mr. Junius S. Morgan to the Council. He is now present at the meeting, and we are all very glad to see him amongst us. He may be assured of the deep veneration in which his name is held both by nurses and by members of the Council. (Hear, hear.) I now beg to move the adoption of the report, the accounts and balance-sheet which have been taken as read. (Applause.)

Mr. NORMAN: I have great pleasure in seconding that.

Mr. KING (consulting actuary): I did not come prepared to make a speech, but I have very great pleasure in saying a few words. These can only be words of congratulation at the great success which has attended the Fund. That success so far has been eminently shown by the accumulation of money, and the balance in hand. This is a very large amount, but, after all, that is not the only object of the Fund. Presently its principal object will be to pay out money, and it has been the object of all concerned to make it so strong and certain that there will never be any doubt as to its being able to pay all that is required. That was one of the great things about it—its great strength. (Hear, hear.) That great strength was not derived by drawing unduly from the members for the simple reason that any surplus there might be will go back to them by bonus. So that every one who takes out a contract with the Fund may be quite certain to get full return for every penny paid in, and that is a point of very great interest to nurses who become policy holders. If they were to join assurance companies, or other societies, a certain proportion of their premiums would be taken for dividends to the shareholders, or in the event of their going to a mutual life insurance company and taking out annuities, a proportion of their payments would go to paying bonus to the policy holders. Therefore, in getting annuities from these companies the nurses would have to pay some part of the profits of others. But in this Fund every penny they pay goes back to themselves, and therefore they cannot do better than join the Royal National Pension Fund. (Hear, hear.) Reference has been made to the quinquennial valuation. At the present time it would be premature for me to speak upon that as the work is not yet finished. The only point that causes any anxiousness is the Sick Pay branch, and that will be thoroughly investigated. There is no reason to fear any financial difficulty, but it may be necessary to revise the table of rates, and put that on a thoroughly satisfactory basis. That is one of the points that will have full attention. After this observation, I have only again to congratulate the Council on the great success attained by the Fund. (Cheers.)

Sir Henry Burdett, who had arrived while Mr. King was speaking, said: I am very sorry indeed that I have been prevented, by circumstances which were unavoidable, from getting here earlier. I intimated to the Secretary this morning that on my return from abroad, I found I was obliged to preside at another meeting. That meeting has been prolonged, and I have only just been able to come to you. I am sure you will understand that it was my misfortune, and not my fault, that has delayed my appearance. Since the last annual meeting we have suffered a grievous loss. Every year since the foundation of this Fund the chair has been taken by the same gentleman, Mr. Burns, and I wish, for my own part, to say only a few words about him, because I believe something has already been said by previous speakers. I cannot allow this occasion to pass without placing on record that after Mr. J. S. Morgan we owe more to Mr. Burns than to anyone else who has been actively associated with the management. Mr. Burns occupied an unique position in the City of London. His death has left a void which all of us who are actively interested in the welfare and business of the City of London realise. Indeed, we do not know how this void can be adequately filled. Those of us who knew Mr. Burns in a more intimate capacity on this Council not only appreciated his great abilities—for in counsel he was a man who stood out above his fellows in an extreme degree—but we recognised in him a man whose sterling worth and whose loyalty were qualities so precious as, in our opinion, to supersede almost those which made him such a power and such an influence in finance in the City of London. To me it has been a great personal loss, for I have lost one of the dearest friends of my life. To the Pension Fund it has been a loss, too, but owing to the very friendships which Mr. Burns was able to form, we are happy in having secured as his successor one of the first of the merchant princes who came forward and subscribed £5,000—with three others making £20,000—which formed the nucleus of this Fund. On the Securities Committee we have a tower of strength in Mr. Hambro, and I have no doubt that the confidence, the deserved confidence, in which he is held by those for whose benefit the Fund has been established, and in the institutions which employ nurses, will result in a more extended business in the future than in the past. There was a vacancy created on the Securities Committee—one of the most important of our committees—by the death of Mr. Burns, and that has been filled by the election of Mr. J. Pierpoint Morgan, jun., the grandson of Mr. J. S. Morgan. Mr. Pierpoint Morgan has only just come to London, but I have had the privilege and happiness of knowing him and his work in the United States. I am glad to see him amongst us, and to know that he possesses the character and ability of his grandfather and father. Whilst, therefore, we welcome him, and benefit by his joining us, we can congratulate the City and the house of J. S. Morgan and Co. in having the benefit of his services as one of the partners in London. (Hear, hear.)

### Sick Pay.

I want particularly to say two things which I do not think Mr. Rawlings mentioned. Although we started the Sick Pay Branch of this Fund with some trepidation, owing to the fact that the actuary's advice was that there were no precise figures upon which to work, still at the conclusion of the second quinquennial period we have arrived at this satisfactory result—that the net sum accumulated in favour of the sick fund and now invested is £1,096. Of course, if we were to leave the sick fund as it is, and Mr. King mentioned the difficulties connected with it, we might possibly find as a result of our experience that our net reserves would not tend to increase, but we are fully alive to the present difficulties, as we were fully alive to the risks when the sick pay branch of the Fund was started. We have had the knowledge and experience of nearly ten years' working among the class, and a special class, for whom we cater. I think, therefore, we may rest confident that with the assistance of our actuary, and in view of the experience we have had of the working of the sick fund we shall be able to make such arrangements as will secure for the future that it shall not only be what it has been in the past—a great consolation and stand-by to the whole nursing body—but from a financial point of view entirely satisfactory because it will be financially sound. We may say we have made a successful experiment, for out of it we have added to our funds a sum of upwards of £1,000; but the experiment shows that some rearrangements and modifications are necessary and desirable. These will now be made, and then not only will the nurses have the increased benefits of new arrangements so far as sick pay is concerned, but the Fund will have the advantage of increased business.

### Free Medical Attendance for Members.

In this connection I have to announce that Dr. Potter, who is our medical officer, in view of the many difficulties which affect nurses when they are sick, has very generously offered to attend without fee any member of the Pension Fund, whether holding a sick-pay policy or not, *i.e.*, all members of the Pension Fund who may call at his consulting-rooms, 8, King Street, Cheapside, E.C. It is felt, from our point of view, that it is of the first importance that our medical officer should have an intimate knowledge, as far as is possible, of every member of our Fund. I do not know how it strikes you, but it strikes me as a very generous act on the part of Dr. Potter that he will afford to the members of our Fund—5,000 in number—opportunity to consult him in order that they may have the advantage of his peculiar experience in relation to the class of diseases and the class of trials which beset nurses, and that is itself of very great importance to nurses, and one which they might be very glad to welcome. (Hear, hear.) I am quite sure that I only give expression to your views and feelings when I express to Dr. Potter our deep sense of indebtedness and our most hearty thanks for his great generosity, for the substantial support which he will give to the Fund, and for the comfort he has placed at the disposal of every member of the nursing profession in the country.

### The Cost of Management.

Nurses do not quite understand the meaning of percentages, and if we say the expenses of 1897 were 3·53 per cent.; therefore, I may say for their benefit that the expenses of management in 1897 only amounted to £3 10s. for every £100 of premium income, including all charges of every kind and description. This should be compared with the average of £4 5s., the average for the last five years. I think that is most satisfactory, and it is certainly satisfactory to you to know that, taking the great mutual and insurance societies of this country, there is no society which conducts its business at anything like so small a rate for management expenses as we conduct ours. (Hear, hear.) This is so, although other societies do much in life insurance, not in annuity and sick pay business, and they will be the first to realise how vast a difference that makes to expenses, having regard to the fact that pensioners have to be dealt with monthly or quarterly, whereas when a life insurance policy has become payable, one item, one entry, and one receipt, disposes of the business. That is not so with us. The nurses require to be paid monthly or quarterly, so that we have extra expenditure in remitting their pensions at the end of the period? That means a very great addition to our expenses. Already £40,000 has been returned to nurses and £5,000 has been distributed in sick pay. £5,400 has been paid in pensions to 141 pensioners, and there is, in addition, a steadily progressive business. I think you will agree that these figures show splendid work, and are proof positive that this fund satisfactorily carries out its objects. There is one item in connection with the benefit branch which I think ought to be mentioned. This year we have had two legacies—the first in the history of the Fund—one by the late Mr. Burns. The other comes to us from an outside source —the executors of the late Mr. Richard Gibbs, who have given £250 to the Benevolent Fund. I can only hope that people who benefit so largely by the services of nurses will bear that fact in mind, and that the example of the late Mr. Richard Gibbs will find many imitators in the future. I have very much pleasure in supporting the adoption of the report.

Election of Policy Holders' Representatives.

The report of the scrutineers of the ballot was read, showing the votes given as follows:—Miss F. C. Nott Bower, 959 votes; Miss Mabel Cave, 939; Miss Mary L. E. Dunn, 949; Miss E. Fisher, 953; Miss L. M. Gordon, 959; Miss K. H. Monk, 952; Miss E. Vincent, 944. In their report the scrutineers said:—"The number of spoiled cards is remarkable, in nearly every case the reason of the votes being rejected being that they contained more than seven names, arising chiefly from the fact that the nurses had repeated in writing one or more names already printed above, although careful directions were given so as to try and avoid such mistakes."

The Chairman declared the seven ladies mentioned duly elected, and the voting cards were ordered to be destroyed.

Mr. Bryant, F.R.C.S.:—I rise to propose a vote of thanks to the Chairman, and I must also include the Deputy-Chairman of to-day (Mr. Rawlings). I need hardly say that we are pleased to see you, Sir (Sir Henry Burdett) in the chair, knowing that you have been such a kind friend to this Fund from the beginning, and the excellent work you have always done for us. (Hear, hear.) We appreciate always your very kind and appreciative words with regard to our late chairman, and also our pleasure in having a Mr. Burns as an active member of this council. We are pleased, too, to have from you such a very encouraging summary of the steady progress of this Fund. I have been associated with it from the very first, and I must admit I had no very sanguine hopes at the outset. But I find its success perfectly astonishing—how steadily, in fact how rapidly, that success has continued to advance. Even now we have not reached the maximum, but we may reasonably look forward to continued success in the future. Knowing something of the troubles of nurses, I can say that this is one of the most valuable institutions ever intended for their benefit.

Mr. King seconded the resolution, and it was carried in a most cordial manner.

Sir Henry Burdett was cheered on rising to respond. In doing so, he thanked those present for the way in which they had passed the vote of thanks. I should like, he continued, to add two facts to what I have already said. First of all, we have established this year a reserve fund which represents the profit on the sale of investments and re-investments, and it commences with a sum of £3,300 to its credit. It is a new feature, and I think an important one, because we have the advantage and have always had the advantage, and, I believe, now that the sons of the gentlemen who founded this fund have joined the Council, we shall continue to have the advantage of the best financial brains in the City in looking after our funds. Still, even the best heads can make mistakes, and it is very important we should have a reserve fund for contingencies. It is satisfactory to know that, having decided upon this step, we have been able to start with £3,300. (Hear, hear.) I should like to say also, as it is a point in which nurses are greatly interested, that the second quinquennial valuation, which is now proceeding, cannot be completed, I understand, until the end of April, and that the nurses must not expect to know the results before May or June. With reference to the benevolent fund, I think there is a point which the public ought to realise, and it is this—nurses are probably the most hard-working of all women-workers, and they surely ought to know better than anybody else how far the benevolent fund is necessary for the requirements of the nurses themselves, and for their protection. It is a remarkable fact that the 5,000 nurses who are members of this fund, and who by their exhibition of providence have provided for themselves, have come forward during the Diamond Jubilee year, and have generously subscribed small sums of one shilling towards the Junius S. Morgan fund, and have added an income of over £100 per annum to that fund. They have already raised no less a sum than £5,000, and the total funds of the benevolent fund now invested are nearly £17,000. This yields an income of £800, of which about £600 are distributed. We have never distributed the whole of the income of the benevolent fund, because we have felt that the demands of the nursing body must tend to increase, and it was essential, as the number of members joining the fund grew larger, that we should have larger reserve funds. The policy of the ladies, who have shown themselves very good women of business, has been to invest a certain proportion of their income, and to meet besides all the really urgent claims made upon them. I think that is a very good policy indeed. I beg to thank you very much for your vote of thanks, and your presence here to-day.

The proceedings then terminated.

### Where to Go.

Royal British Nursing Association.—At 11, Chandos Street, Cavendish Square, the fourth sessional lecture will be given by Miss Lilias Hamilton, M.D. (late physician to the Ameer), on Friday, the 25th inst., at 8 p.m. The subject will be "Afghanistan," and will be illustrated by lantern slides.

The Dowdeswell Galleries, 160, New Bond Street.—A charming series of coast scenes is now being exhibited at the Dowdeswell Galleries by W. Wyllie, A.R.A. Mr. Wyllie has always been a great favourite as a painter of marine subjects, and the collection which is now on view shows how completely the artist understands the varying moods of sea and sky. It is a great pleasure to have an opportunity of viewing such a charming collection pictures.

### Presentations.

Dr. Adams, who for some time has been house-surgeon at the Dorset County Hospital, has been presented with various tokens of good-will from his fellow-workers and patients. Dr. Lush gave him a valuable book; the hon. surgeons, a photograph of the hospital; the nursing staff, a silver ink-stand; the dispenser and male patients, a tobacco jar and cigar holder. Dr. Adams is leaving Dorchester for work in London.

### The Prince of Wales's Hospital Fund.

### THE PRINCESS'S LETTER.

In no movement yet set on foot have all the Royal family united in taking a greater personal interest than in the Prince of Wales's Fund. This fund was suggested by the Prince to the people of England, and more especially to the people of London, as the manner most pleasing to the Queen for the commemoration of her long and prosperous reign by her subjects. A desire to give pleasure to the Sovereign whose life has been devoted to the service of her people was to bring help and
healing to the sick
bed of their poor
afflicted brothers &
sisters.

Alexandra

Princess of Wales

Sandringham
Norfolk.

It is very near to my heart, that the

Prince of Wales

Hospital Fund in commemoration of the Queen's Diamond

Jubilee, should be a perfect and permanent success

I therefore now appeal–

PORTION OF THE LETTER WRITTEN BY THE PRINCESS OF WALES TO THE CHILDREN OF ENGLAND.

naturally their one aim and object in any act of commemoration. The generosity with which the Prince's suggestion has been responded to must have gratified the Queen, and it will, we feel sure, be a source of future regret if there is one of her subjects in the metropolis who does not join hands with the loyal citizens in an act of recognition of the devotion to the nation which she has exhibited. It has been felt by many who have not large sums to give that a small contribution is too insignificant to offer. But on the small, as well as the great contributions, the success of the fund depends, and unless it is representative of the feeling of each and every dweller in the metropolis at least, it will not have fulfilled its object.

### A ROYAL EXAMPLE.

It was more especially to emphasise this desirable unity that the Princess's letter, of which part is reproduced in our columns, was written. It is hoped to win the co-operation not of one member but of every member of every family in the good work of providing for the poor when illness overtakes them. The Prince and his family set the first example of this unity of purpose. The Duke of York has freely given of his service and his interest, and the little Prince Edward was one of the first little children who became an annual supporter of the hospitals under the Prince's scheme. All the Royal Children of England have joined the Great Roll of Ministering Children. With the hope of inducing mothers and fathers and their children to follow the Royal example, the Princess has written the charming letter with her own hand, addressed to the children of England, and attached to every album issued to contain and preserve the Hospital Stamps. The love which the Princess bears to children is well known, and is especially illustrated by her frequent visits to children's hospitals, where her warm sympathy with the little sufferers is ever apparent. It is therefore especially appropriate that the Princess should address the youthful portion of the community, and use all her influence to induce them to commence a good work, which will provide an increasing interest throughout their lives.

A GREAT ROLL FOR ADULTS.

But, welcome as will be all children to increase the volume of the great roll, it is desired to enlist all adults also who may only be able to give a small yearly gift. The Hospital Stamp scheme has been especially designed to assist the annual subscribers of small amounts, and especially is it hoped that all those who belong to institutions, societies, guilds, and other communities, will come forward also and enrol themselves. For instance, here in THE HOSPITAL we might appropriately point out to medical students and nurses that they could be enrolled under the name of the hospital at which they are working, and that a page of the Great Roll now in preparation can be filled by one hundred portraits. Messrs. Martin and Snallnow, of 416, Strand, are prepared to take these photographs of adults, free of cost, just as Messrs. Speaight, of 178, Regent Street, are arranging in respect to young subscribers. The stamp albums and the stamps can be obtained at the photographers, or can be ordered from any stationer, bookseller, or chemist. Those who have already subscribed can still help by showing others how to join the movement. Those in charge of the Fund have spared no pains to offer the best facilities for every class of subscriber to honour the Queen, and help the hospitals through the medium of the stamp scheme.

### What is Massage?

BY A HOSPITAL SISTER.

### II.—MASSAGE AS APPLIED TO MEDICAL AND SURGICAL CASES.

(*Concluded from page* 220.)

FOR many years massage was employed in this country as a means of reducing weight. "A force de bras" was the chief factor in this mode of treatment, and the masseuse did not require much technical skill, and received little or no theoretical training. The next step in its evolution was the introduction of the "Weir-Mitchell system," the value of which is well known in nervous complaints. Latterly, massage has been used for many other diseases, and its most recent development has been its application to various surgical cases. It will be readily seen that a masseuse undertaking such delicate and complicated work must be well grounded in the elements of anatomy and physiology. It is in this respect that the Swedish training is so infinitely superior, with its practical demonstrations in anatomy, to that obtainable in other countries. One of the best proofs of the acknowledged value of treatment by massage is the fact that it is now employed in many of the London hospitals in cases of fracture, &c. It will doubtless be of interest to note the following instances of different cases where massage has proved successful Constitutional diseases, such as anemia, general debility, &c., joint diseases, rheumatism, gout, synovitis; also cases of fracture, sprain, nervous diseases, sciatica, writer's cramp, neuralgia, insomnia, &c. It will be observed that the foregoing are all more or less chronic complaints, but massage may also be applied with great advantage in the convalescent stage of acute disease. Perhaps its greatest triumph has been achieved in the treatment of spinal curvature, where it has been combined with Swedish curative exercises. The chief reason that massage has not sooner obtained a definite status in England is owing to incompetent workers. When this objection is removed we trust it will occupy the same high position that it does in other countries.

### Appointments.

### MATRONS.

DUNSTER AND MINEHEAD VILLAGE HOSPITAL.—Miss Mary Machen has been appointed Matron of the Dunster and Minehead Village Hospital. Miss Machen was trained at St. Mary's, Paddington, and afterwards took night superintendent's duty there. In 1884 she was one of the four nurses selected by Her Royal Highness the Princess of Wales for service in Egypt. She has also worked as sister at the Hospital for Sick Children, Great Ormond Street, and at the Hospital for Consumption, Brompton. For the last three years and a-half she has been matron of the small Memorial Hospital, Almondsbury.

ASSOCIATION FOR THE RELIEF OF INCURABLES IN GLASGOW AND THE WEST OF SCOTLAND, GLASGOW.—Miss Margaret Whitecross has been elected to the post of Matron in the Broomhill Home for Incurables, Kirkintilloch, near Glasgow. Miss Whitecross received her training as nurse in the Western Infirmary, Glasgow, and afterwards was sister in the Victoria Infirmary, Glasgow. In 1892 she was appointed sister in charge of four wards in the Western Infirmary, and at present holds the appointment of assistant-matron in that institution.

SIMMER AND JACK HOSPITAL, JOHANNESBURG.—The Matron appointed to this hospital in February last is Miss Kate Gossage. Miss Gossage's record is as follows: She was trained and for five years in the wards and on the private staff of Guy's; charge nurse at the North-Eastern Fever Hospital, London; and sister of the women and children's wards, Swansea Hospital, which she left last October. Her present appointment is to the largest hospital on the gold reef of South Africa.

CHARNWOOD FOREST CONVALESCENT HOME, LEICESTERSHIRE (CHILDREN'S BRANCH).—Miss Strong has been appointed Matron of this Home, about to be opened for children. Miss Strong was trained at the Leicester Infirmary, where she has been working for several years. She has held the post of matron of Lady Salt's Convalescent Home for Children at Telford, Surrey, and has been sister of the children's medical ward at the Leicester Infirmary for three years.

TIVERTON INFIRMARY AND DISPENSARY.—On March 16th Miss Annie Phillips was elected Matron of the above infirmary. Trained at King's College Hospital, sister at the Orthopædic Hospital, Great Portland Street, and sister at the Victoria Hospital for Children, Chelsea, Miss Phillips has good credentials for her new office.

FOUNTAIN FEVER HOSPITAL, LOWER TOOTING.—Miss Kathleen Lucretia Burleigh was appointed Matron of this hospital on March 12th, 1898. She was probationer for three years, staff nurse for one year, acting night superintendent and assistant-housekeeper successively at St. Bartholomew's Hospital, London.

### The Children's Nurses Again.

FROM an article in the *Queen* for the 12th inst. we learn that there is a great demand for children's nurses in the Northern Provinces of India, scarcely any for sick nurses, and none at all for typewriters, secretaries, or companions. The reasons for this are not far to seek. Anglo-Indians are as rule poor, they cannot afford luxuries. An increased knowledge of hygiene and sanitation has lifted the trustworthy English nurse out of the requirements that rank as luxuries among those which come under the heading of necessities. The advantages offered are not great. Salaries run from £20 to £25; the position is rather better than that of a governess in England, because the English nurse is a "sahib"; but the life is dull, and few of the social distractions enjoyed by her employers filter to the nursery, whilst a lady would have to associate with women of a lower class who hold the same position as herself, namely, that of a children's nurse. Still the demand is very great, and a strong, capable woman, could soon make her value felt, and would never lack employment.

### Everybody's Opinion.

**[Correspondence on all subjects is invited, but we cannot in any way be responsible for the opinions expressed by our correspondents. No communication can be entertained if the name and address of the correspondent is not given, as a guarantee of good faith but not necessarily for publication, or unless one side of the paper only is written on.]**

### NURSES' AMBITIONS.

"NURSE-MATRON" writes: In answer to "I Spy's" letter in THE HOSPITAL of March 19th, I will give my experience. I entered a large provincial hospital, at the limit of age when probationers are usually received, with a definite object in view, namely, that of becoming a school matron. I enjoyed the work and did not find it overtaxed me, never being off duty for illness all the time I was there, as the most of the younger pros. were. The Matron, who knew my views, kindly arranged for me to spend the greater part of my time in the male wards. Provided with my certificate and an excellent private testimonial from the Matron, I soon succeeded in finding the post I had set my heart on, and am now nurse-matron in the preparatory house of one of the oldest public schools in England. I am happy in my work and in the affection and confidence shown me by these young boys, for whose souls and bodies I am in such a large measure responsible. I agree with "I Spy," that if people knew what they wanted and kept that aim steadily in view, there would be fewer failures.

### HOW TO START A DISTRICT NURSE.

"L. M." writes: Seeing in THE HOSPITAL, under "Notes and Queries," that "E. A. B." wishes for suggestions as to how to start district country nursing, it came to me that perhaps a few thoughts from one who has done a good deal of district nursing might be useful. I started district midwifery in L——, including the extended borough, which was often quite in the country. The usual way to begin is to form a committee and then get promises of annual subscriptions so as to guarantee the nurses' salaries. It might be possible to get a grant from the guardians on condition that the parish doctor might have the services of a nurse in special cases if he wanted her. If midwifery is to be carried on, I should suggest it being kept quite separate from the general nursing. For my own part, I always think it more satisfactory to give the nurse a salary, and let it be inclusive. Of course, in a country district, board and lodging is cheaper than in a town; in the latter, £80 inclusive, is an ordinary salary, but where there is a Young Women's Christian Association, one could manage with a little less. Where two nurses are required to work together, it works better, and things seem to fit more easily if the two have a previous knowledge of each other; in short, are "friends." I shall be pleased to answer any questions in detail as to how district nursing is worked, should your correspondent wish for them.

### Novelties for Nurses.

### A NEW HAIR INVIGORATOR.

WE have received a sample of a new hair wash from the 4,711 Eau de Cologne Depôts at 62, New Bond Street. This hair-wash, called Captol, is most refreshing and cleansing, and far less sticky or greasy than many similar preparations. Captol is claimed to be an unfailing remedy in all cases of premature baldness, eradicating dandriff with greater celerity than other preparations. It has been prepared at the suggestion of Dr. Eichoff, of Elberfeld, and this in itself is in favour of its usefulness for the purposes indicated. It is an alcoholic preparation of a chemical compound, the constituents of which have long been found efficacious as a hair stimulant, whilst the chemical combination as in Captol, is said to be effective without any irritating action. Captol is most agreeably perfumed, and contains no inflammable substance like petroleum, and is free from lead compounds.

### Notes and Queries.

*How to Fill a Water-Bed and to Make a Linseed Poultice.*

(217) "Ignoramus" would be very glad to know (1) the test for knowing when a water bed is full enough and how to fill them; (2) how to make a mustard and linseed poultice.

1. Fill a water bed with warm water until there is enough to prevent the top touching the underneath when the patient is on it. This can easily be ascertained by slipping the hand beneath bed and patient; if there is then a layer of water between the patient and the hand the amount is right. 2. Heat a basin, partly fill with boiling water, and quickly sprinkle in enough linseed meal, whilst stirring briskly, to make a smooth, soft, but not sloppy mass. Spread on flannel or muslin, turn up edges all round, smear with a little sweet oil or cover with a layer of muslin, and apply. The mustard may either be sprinkled on the surface or mixed with the meal. Half a teaspoonful of mustard to one tablespoonful of linseed meal is a useful proportion.

*Training for the Holt-Ockley.*

(218) A domestic servant, 23 years of age, extremely fond of nursing, wishes to know if she would be a suitable candidate for the Holt-Ockley system of nursing? (2) Where she could train and what she would have to pay?—*S. M. P.*

This girl appears to be of the right stuff of which to make a good district nurse, and we advise her to apply to Queen Victoria's Jubilee Institute for Nurses, 29, Castle Terrace, Edinburgh. The council pay for the training of a certain number of probationers (amongst whom may occasionally be found a domestic if suitable in other respects), and also pay the probationers a salary of £5 for the first year and £10 for the second during training. She will then be a Queen's Nurse, which is a higher position than that given by the Holt-Ockley.

*Musical Instruments.*

(219) What is a suitable musical instrument for a nurse to learn? Is a harp difficult and expensive?—*G. S.*

A harp is very expensive, and would be cumbersome to move from place to place. If "G. S." has anything of a voice the guitar is very sweet and suitable. The violin—the prince of instruments—is also easily carried about, but it is difficult to learn, and somewhat of a trial to the neighbours during the process.

*Nightingale.*

(220) "Not a Nurse" wishes to know what is meant by a "Nightingale" jacket, and how to make one.

Take from a yard and a half to two yards of flannel. Halve it, and cut a slit five inches deep from one selvedge. Bind all round—round the slit as well—and turn the two corners of it back. This is for the neck. Turn up the two bottom corners for five inches, so that the arms may be slipped through, and thus keep the wrap in place.

*Who Owns the Uniform?*

(221) I have been a district nurse for 12 months. Who owns the uniform I have worn when I leave?—*Bess.*

"Bess" must send name and address before we can answer her query.

*Measles.*

(222) 1. Can measles be carried by a third person? 2. How long does the infection last? 3. What is necessary in the way of disinfection of persons, clothing, &c.?—*No. 2,295.*

1. Measles can be carried by third persons, but probably is very seldom so carried unless the person in question has had pretty close or prolonged exposure to the infection. For example, a nurse after her visit would be more likely to carry it than a doctor after his. 2. In "Twentieth Century Practice" Dr. Dawson Williams says that the "period of isolation should extend to three weeks at least, and the patient should be free from all desquamation and cough before being allowed to mix with susceptible children." In regard to children who may have been exposed to infection, he says: "The period of observation recommended by the Clinical Societies' Report is fourteen days, and the individual must be at the completion of that time free from fever or catarrh. The Code fixes sixteen days, which is safer, as it would include nearly all even of the most exceptional cases on record." 3. Washing patient, boiling linen, baking or steaming clothes, washing room.

*Zenana Work.*

(223) A trained nurse holding L.O.S. certificate wants to know how to obtain work in Indian zenana.—*H. B.*

Only as a missionary. This work is done principally by native women trained by the different medical charities.

*A Nurses' Club.*

(224) Is there a nurses' club where nurses for a small sum could see the *Lancet* and *British Medical Journal* regularly?—*Nil Desperandum.*

Great Ormond Street is near the British Museum. By getting a householder to sign a request for admission to the reading-room "Nil Desperandum" could see these papers free. Also the free library nearest her will probably have it on the table. She can also obtain them from some circulating libraries for 2d. a copy for home reading for a few days.

*Nurses' Home in London.*

( ) I am going for monthly training to a lying-in hospital. Where can I get cheap board and lodging?—*Dorothy.*

The Nurses' Hostel, Francis Street, W.C. Cubicle and full board, 17s. 6d. a week might suit you, at any rate for a time. You do not say where your training school is, and you must be near it.

*Nursing in Ireland.*

Nurse Ireland must send her name and address before we can accede to her request.

## Figures and Tables

**Figure f1:**